# The value of chromosome instability detected by low−pass whole−genome sequencing in preoperative prediction of sentinel lymph node metastasis in breast cancer

**DOI:** 10.3389/fonc.2024.1434526

**Published:** 2024-10-04

**Authors:** Jian Zheng, Fen Xu, Guangying Li, Moubin Lin, Hua Hao

**Affiliations:** ^1^ Department of Pathology, Yangpu Hospital, School of Medicine, Tongji University, Shanghai, China; ^2^ Department of General Medicine, Yangpu Hospital, School of Medicine, Tongji University, Shanghai, China; ^3^ Department of General Surgery, Yangpu Hospital, School of Medicine, Tongji University, Shanghai, China

**Keywords:** chromosomal instability, low-pass whole-genome sequencing, invasive breast cancer, sentinel lymph node metastasis, receiver operating characteristics

## Abstract

**Background:**

Breast cancer is a malignancy characterized by chromosomal instability (CIN). This study aimed to examine the potential diagnostic value of chromosomal instability, detected by low-pass whole-genome sequencing (LPWGS), in the preoperative evaluation of sentinel lymph node metastasis (SLNM) in breast cancer.

**Methods:**

A retrospective investigation of clinical records from 29 patients with breast cancer revealed two distinct groups based on sentinel lymph node biopsy (SLNB) results: the SLN metastasis group (24 cases) and the SLN non-metastasis group (five cases). CIN and CIN scores were evaluated using LPWGS. An analysis of univariate data and binary logistic regression was employed to identify factors influencing SLNM, and a curve with receiver operating characteristics (ROC) was constructed to assess the diagnostic utility of CIN in predicting SLNM.

**Results:**

A significant association between the SLNM and CIN high groups was observed in breast cancer (*P*=0.011). The CIN score in the metastasis group (17,665.055 ± 8,630.691) was higher than that in the non-metastasis group (9,247.973 ± 3,692.873), demonstrating a significant difference (*P*=0.044). Univariate binary logistic regression analysis indicated that CIN was a significant predictor for SLNM (odds ratio: 4.036, 95% CI: 1.015–16.047, *P*=0.048). The AUC of CIN for preoperative diagnosis of SLNM was 0.808 (95%CI: 0.635–0.982, *P*=0.033), with a sensitivity value of 67.0% and specificity of 100.0% at a threshold of 13,563.

**Conclusion:**

Detecting CIN through LPWGS demonstrates diagnostic potential in predicting SLNM in patients with breast cancer before surgery. This approach offers a novel method for assessing axillary lymph node status in clinical practice.

## Introduction

The prevalence of breast cancer has steadily increased over the past few years. In 2020, breast cancer has become the most common type of cancer, surpassing lung cancer, posing a substantial threat to women’s physical and mental well-being (1). The preoperative assessment of axillary lymph node (ALN) status plays a critical role in determining the breast cancer stage and guiding surgical interventions. The sentinel lymph node (SLN), identified as the initial or group 1 lymph node through which breast cancer must pass to metastasize to the ALN, is particularly crucial for this assessment. The incidence of skip metastasis in axillary lymph nodes is infrequent. Thus, SLN metastasis (SLNM) can provide more accurate predictions regarding the condition of additional lymph nodes (2, 3). Currently, SLN biopsy (SLNB) has emerged as the primary procedure for determining ALN status in patients with breast cancer (4). Frozen sections or cell blots are often used to detect SLNM, but they have low sensitivity for identifying micrometastases (5). Paraffin-embedded tissue sections are the standard for assessing SLN status, but this process is lengthy, typically taking 2–5 days to release the report. This delay can result in increased medical costs and patient anxiety, and some patients may require secondary surgery (6). Hence, it is imperative to thoroughly investigate the predictive and evaluative framework of SLNM in breast cancer before surgery.

Chromosomal instability (CIN), characterized by heightened occurrences of chromosomal gains or losses during segregation, leads to alterations in chromosome count and structure (7). Cancer cells exhibiting CIN demonstrate enhanced adaptability and metastatic potential (8). CIN is more prevalent in metastatic breast cancer than in primary breast cancer (9), underscoring its association with metastasis (10). Recently, next-generation sequencing (NGS) has established itself as a viable method for assessing CIN, with low-pass whole-genome sequencing (LPWGS) being incredibly efficient in capturing comprehensive genomic changes at a relatively low cost. Zhu et al. (11) used LPWGS to detect gene changes in formalin-fixed, paraffin-embedded (FFPE) samples and found that high CIN was linked to TP53 copy loss and poor prognosis in BRCA1 germline-mutated breast cancer. In addition, Fitzpatrick et al. (12) showed that ultra-LPWGS technology can detect circulating tumor DNA in the cerebrospinal fluid of patients with breast cancer and leptomeningeal metastasis, allowing for accurate evaluation of treatment response.

Recent research predominantly investigates mechanisms underlying CIN and its impact on the initiation, progression, treatment, and prognosis of breast cancer (7, 11, 13–20). While existing evidence has demonstrated a significant correlation between CIN and tumor metastasis (21), with research predominantly concentrating on elucidating the molecular mechanisms by which CIN facilitates cancer cell metastasis (7, 22, 23), clinical studies investigating CIN as a predictor of SLNM remain limited. Therefore, the objective of this research is to assess the precision and effectiveness of CIN detection through LPWGS as a means of predicting SLNM preoperatively in patients with breast cancer. We hope this investigation can offer clinicians a novel and dependable preoperative diagnostic approach, thereby enhancing disease assessment and ultimately improving patient outcomes and prognosis.

## Methods and materials

### Study population and samples

**Figure 1:**
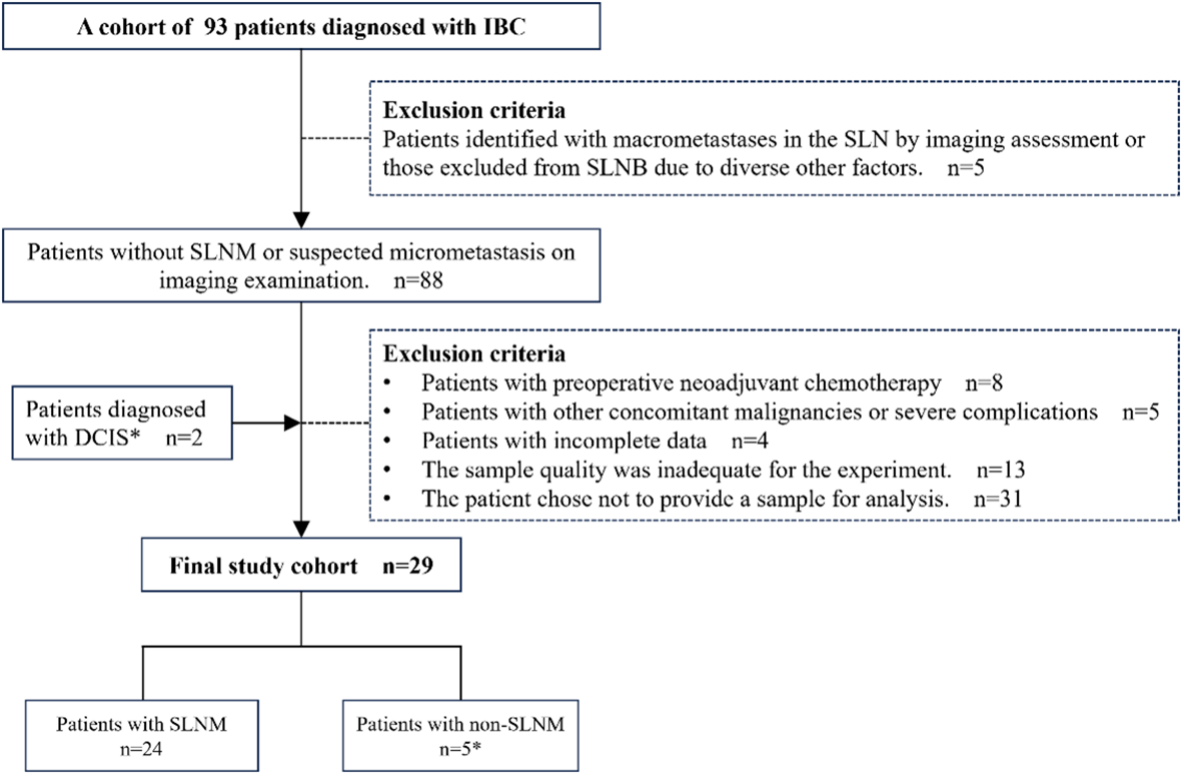
Patient selection overview. IBC, Invasive breast cancer; DCIS, Ductal carcinoma *in situ*; SLNM, Sentinel lymph node metastasis; SLNB, Sentinel lymph node biopsy. *Two patients with DCIS were included for the following reason: DCIS is intricately associated with IBC and may serve as a precursor to invasive malignancies, occasionally co-existing with IBC (24–26). Despite therapeutic interventions, a subset of patients with DCIS eventually progress to invasive disease (26). Therefore, incorporating DCIS cases into research facilitates a more comprehensive understanding of CIN across various stages of breast cancer, thereby enhancing the representativeness of the study and the robustness of the statistical analysis.

A retrospective analysis of clinical data was conducted on 93 patients with invasive breast cancer (IBC) admitted to Yangpu Hospital Affiliated to Tongji University (Shanghai Yangpu District Central Hospital). The inclusion criteria required the absence of SLNM or suspected micrometastases on initial imaging, as well as a pathologically confirmed diagnosis of IBC. The exclusion criteria encompassed the following: patients with SLN macrometastasis identified through imaging studies or those who did not undergo SLNB due to other factors; patients who had undergone neoadjuvant chemotherapy prior to surgery; patients with concurrent malignancies or severe complications; patients with incomplete data; and patients who declined participation in the study, or whose samples failed to meet the requisite quality standards. Furthermore, given the potential progression of ductal carcinoma *in situ* (DCIS) to IBC (24–26), this study incorporated two cases of DCIS in the final analysis to augment the representativeness and robustness of the statistical findings. Following a thorough evaluation of the clinical data and sample quality from all patients, the samples from 29 patients were ultimately selected for inclusion in this study for subsequent CIN detection and analysis (**Figure 1**). This study received approval from the Ethics Committee of Yangpu Hospital Affiliated to Tongji University (Approval code: LL-2021-WSJ-010), and all patients provided written informed permission.

### Collection of clinical and pathological data

The clinical data and postoperative pathological reports of all patients were collected, including age at diagnosis, histological type, histological grade of invasive ductal carcinoma, molecular subtype, estrogen receptor (ER) status, progesterone receptor (PR) status, HER2 and Ki67 status, surgical methods, and postoperative treatment. The status of patients with HER2 score of “2+” was determined according to the results of fluorescence *in situ* hybridization in clinical data. After examining the hematoxylin and eosin slides, FFPE tissue samples were collected from the patients for further DNA extraction, sequencing, and chromosomal instability analysis.

### Genomic DNA extraction and library construction

Genomic DNA (gDNA) was extracted from the sample utilizing the Amp Genomic DNA Kit (QIAGEN), following the manufacturer’s protocol. The extracted gDNA sample must adhere to the following criteria: a total quantity exceeding 10 ng, a volume of no less than 20μL, and preservation of integrity with minimal degradation. A certain amount (5ng-50ng) of gDNA samples was enzymatically fragmented using the KAPA DNA Enzymatic Fragmentation Kit to produce DNA fragments with an average length of 300 base pairs (bp). The enzymatic digestion was performed following the manufacturer’s protocol to ensure consistent and accurate fragment sizes.

The fragmented DNA was then constructed into a Library suitable for high-throughput sequencing using the NEBnext Ultra II DNA Library Prep Kit for end repair, splice, and amplification. The finalized libraries were subjected to rigorous quality control measures, encompassing, but not limited to, the determination of DNA concentration, analysis of fragment size distribution, and assessment of library diversity.

### Sequencing data processing and quality control

Approximately 10 million DNA sequences of varying lengths were acquired through DNA sequencing. The raw image data generated by the Illumina X10 platform were subsequently processed through base calling to convert them into serial data, referred to as raw reads. Before proceeding with further analysis, a series of rigorous quality control measures were applied to the original read data. Initially, reads containing joint sequences were filtered out. Subsequently, paired reads were discarded if the proportion of ‘N’ bases in single-end sequencing reads exceeded 10% of the read length. Lastly, paired reads were removed if the number of low-quality bases (quality score less than 5) in single-end sequencing reads surpassed 50% of the read length. The quality control datasets were subjected to statistical analysis and sequence alignment. The statistical parameters evaluated included read count, data yield, sequencing error rate, Q20 content (indicating base accuracy greater than 95%), Q30 content (indicating base accuracy greater than 99.9%), GC content, among others.

The initial alignment results, in BAM format, were generated by mapping against the human reference genome (http://hgdownload.soe.ucsc.edu/goldenPath/hg19/bigZips/) utilizing BWA (http://bio-bwa.sourceforge.net/bwa.shtml). Subsequently, the alignment data were sorted using SAMtools (http://samtools.sourceforge.net/), followed by the marking of duplicate reads with Picard (http://picard.sourceforge.net/). Finally, the post-processed alignment results, with duplicates labeled, were employed for statistical analyses, including coverage and depth assessments.

### Analysis of chromosome instability identified by LPWGS

The cyclic binary segmentation (CBS) algorithm, implemented in the R package DNAcopy (11, 27), was utilized to identify significant genomic breakpoints and segments exhibiting copy number variations. For each sample, a minimum of 10 million paired-end reads were collected. These readings were compared to the human reference genome hg19 by BWA, and the average coverage per 200 kilobase pair dataset of the genome was determined utilizing the Samtools mpileup software (11, 27). The average coverage across all data sets was normalized using Equation (1), yielding the Z-score, which serves as a metric for assessing the stability of individual chromosomes. A Z-score exceeding 3 indicates amplification, whereas a Z-score below 3 suggests deletion.


(1) $$coverage_{normalized}=\frac{coverage_{raw}-mean\left(coverage_{controls,\;raw}\right)}{stdev\left(coverage_{controls,raw}\right)}$$

Equation (2) was employed to quantify the extent of variation across the entire genome relative to the human reference genome, resulting in the calculation of the CIN score. This score was subsequently utilized to assess the stability of all chromosomes. L_chr_ represents the chromosomal segment length, and Z_chr_ represents the Z-score of the chromosomal segment.


(2) $$CIN_{score}=\sum_{all\;chr}L_{chr}*Z_{chr}$$

### Statistical analysis

Statistical analysis was accomplished utilizing IBM SPSS version 27.0 and GraphPad Prism version 9.0. Measurement data following anormal distribution were presented as mean ± standard deviation ( $\bar{x}$ ± s), while count data were represented as numerical values and percentages. The comparison of measurement data between the two groups was performed using a t-test for independent samples. Fisher’s exact test was utilized to analyze categorical variables, and Cramer’s V was employed to evaluate the correlation strength and direction between two categorical variables. Variables with a P value less than 0.05 in the analysis above were considered in a binary logistic regression analysis to investigate the determinants influencing SLNM in breast cancer. A receiver operating characteristic (ROC) curve was constructed, and the area under the curve (AUC), along with a 95% confidence interval (CI), was computed to assess the predictive capability of CIN in identifying SLNM in breast cancer. *P*< 0.05 was considered statistically significant.

## Results

### Overview of patients’ clinical data and general information

This study included 29 subjects, and the clinicopathological information and molecular profiling results for each patient are presented in **Table 1**. The age range of the patients at diagnosis was 30 to 81 years, with an average tumor size of 2.220cm. The primary pathological types observed were IBC and DCIS. The former included specific subtypes, such as invasive ductal carcinoma, invasive lobular carcinoma, invasive micropapillary carcinoma, invasive carcinoma with neuroendocrine differentiation, and invasive breast cancer with medullary features. Out of the 29 patients, 24 examined positive for ER, 22 examined positive for PR, and three examined positive for HER2. All patients underwent SLNB during the operation. Based on the biopsy results, the patients were categorized into a metastasis group (SLNM, 24 cases) and a non-metastasis group (SLN without metastasis, five cases). The selection of appropriate postoperative adjuvant treatment was determined by the examination outcomes and willingness of the patients.

**Table 1:** Clinical and pathological information of 29 patients.

Sample ID	Age of diagnosis	Tumor size (cm)	Histologic type^a^	Histologic grade	ER	PR	HER2	Ki67	Molecular type^b^	Surgical approach^c^	SLNM	CIN score	Postoperative Treatment^d^
1	64	1.6	IDC	I	+	+	–	<14%	Luminal A	MRM	Yes	Low	CT
2	66	3.0	IDC	II	+	+	+	<14%	Luminal B	BCS; ALND	Yes	High	CT
3	40	1.3	IDC	III	–	–	–	>30%	TNBC	BCS; ALND	Yes	High	CT
4	46	1.5	IDC	II	+	+	–	>14%	Luminal B	BCS	Yes	High	No
5	68	2.0	IDC	II	+	+	–	<14%	Luminal A	SM	Yes	High	CT
6	30	1.2	ILC	Other	+	+	–	>30%	Luminal B	BCS	Yes	High	CT
7	64	4.0	IDC	II	+	–	–	>14%	Luminal B	MRM	Yes	High	CT
8	79	2.6	ILC	Other	+	+	–	<14%	Luminal A	SM; ALND	Yes	High	ET
9	37	5.0	IDC	II	+	+	–	>30%	Luminal B	SM; ALND	Yes	Low	CT
10	80	1.6	IDC	I	+	+	–	<14%	Luminal A	BCS	Yes	High	ET
11	61	1.5	IDC	I	+	+	–	<14%	Luminal A	BCS	Yes	High	CT
12	44	5.0	IMC	Other	+	+	–	>14%	Luminal B	BCS; ALND	Yes	Low	CT
13	58	1.0	IDC	II	+	+	–	>20%	Luminal B	SM; ALND	Yes	High	CT+RT
14	49	1.5	IDC	II	+	+	–	<14%	Luminal A	Lumpectomy	Yes	Low	CT
15	72	1.8	IDC	II	+	+	–	<14%	Luminal A	MRM	Yes	High	ET
16	73	2.2	IDC	II	+	+	–	>14%	Luminal B	SM; ALND	Yes	Low	ET
17	64	2.5	NC	Other	+	+	–	<14%	Luminal A	MRM	Yes	High	CT+ET
18	69	2.2	IDC	II	–	–	+	70%	Over-HER2	BCS; ALND	Yes	High	CT
19	81	1.5	IDC	I	+	+	–	15%	Luminal B	BCS	Yes	Low	ET+RT
20	73	3.0	IDC	II	+	+	+	15%	Luminal B	SM; ALND	Yes	High	CT
21	54	3.5	IDC	III	–	–	–	65%	TNBC	SM; ALND	Yes	Low	CT
22	51	3.5	IDC	II	+	+	–	12%	Luminal A	BCS; ALND	Yes	Low	CT
23	71	1.5	ILC	Other	+	+	–	8%	Luminal A	BCS; ALND	Yes	High	ET+RT
24	71	1.5	IMC	Other	+	+	–	5%	Luminal A	BCS; ALND	Yes	High	CT
25	68	0.9	DCIS	Other	+	+	–	5%	Luminal A	BCS	No	Low	NA
26	80	1.5	IDC	I	+	+	–	15%	Luminal B	BCS	No	Low	NA
27	69	2.0	IDC	II	+	–	–	8%	Luminal B	SM	No	Low	CT
28	75	1.5	DCIS	Other	–	–	–	3%	TNBC	SM	No	Low	No
29	40	2.5	IBCM	Other	–	–	–	80%	TNBC	BCS	No	Low	CT

a Histologic type: DCIS, Ductal carcinoma in situ; IDC, Invasive ductal carcinoma; NC, Neuroendocrine carcinoma(Mainly invasive); ILC, Invasive lobular carcinoma; IMC, Invasive micropapillary carcinoma; IBCM, Invasive breast cancer with myeloid features.

b Molecular type: TNBC, Triple Negative Breast Cancer; Over-HER2, HER2 overexpression.

c Surgical approach: MRM, Modified radical mastectomy; BCS, Breast-conserving surgery; SM, Simple mastectomy; ALND, Axillary lymph node dissection.

SLNM, Sentinel lymph node metastasis.

d Postoperative Treatment: CT, Chemotherapy; ET, Endocrine therapy; RT, Radiation therapy.

NA, Not available; ER, Estrogen receptor; PR, Progesterone receptor; HER2, Human epidermal growth factor receptor 2.

### CIN and its score in 29 breast cancer patients

**Figure 2:**
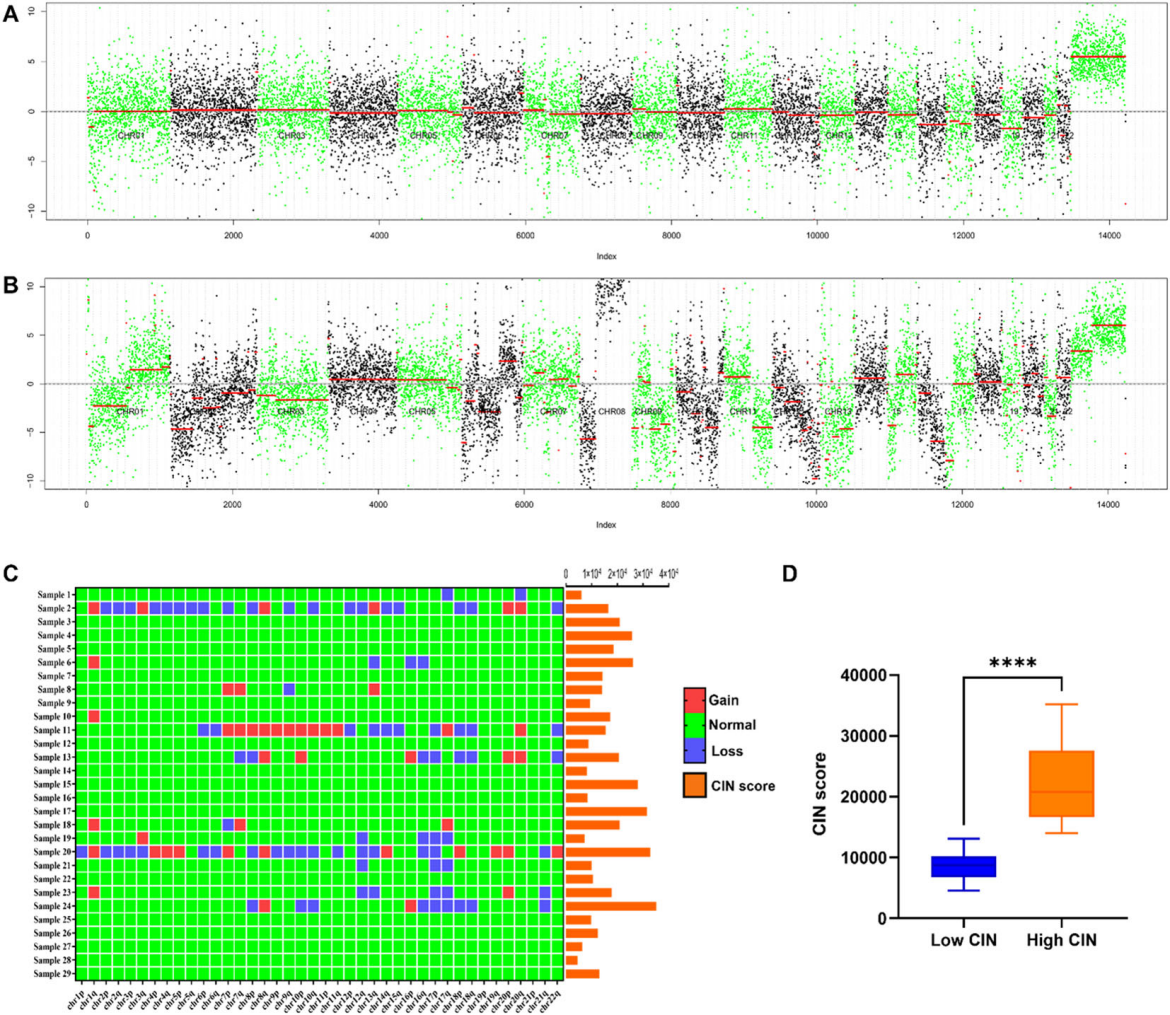
**(A)** Chromosome copy number coverage chart in the non-metastatic group. The non-metastatic group did not exhibit significant chromosomal alterations. **(B)** Chromosome copy number coverage chart in the metastatic group. Chromosomal aberrations were more frequently observed in the samples from the metastatic group as compared to the non-metastatic group. Notably, recurrent aberrations were identified on chromosomes 1, 7, 8, 12, 13, 16, 17, 18, and 20. In the copy number coverage chart, the alternating black and green colors facilitate the differentiation between various chromosomes, while the red line indicates the median value. **(C)** CIN score and chromosome arm changes of 29 patients. Chromosomal regions 1q and 8q exhibited a higher incidence of gains, whereas regions 12q, 16q, 17p, and 17q demonstrated a greater frequency of losses. **(D)** Comparison of CIN scores between CIN high group and CIN low group. ****P < 0.0001.

In the metastatic group, significant chromosomal copy number variations are highlighted when comparing the total normalized coverage per 200 kilobase bin in FFPE samples from patients to those in the non-metastatic group, as illustrated in **Figures 2A, B**. Notably, recurrent aberrations were identified on chromosomes 1, 7, 8, 12, 13, 16, 17, 18, and 20. Additionally, **Figure 2C** provides a schematic representation of CIN scores and changes in chromosome arms, with chromosomes 1q and 8q showing more frequent gains and chromosomes 12q, 16q, 17p, and 17q showing more frequent losses. This phenomenon may be associated with CIN signatures, including chromothripsis amplification, loss of heterozygosity (LOH) (28), and homologous recombination deficiency (29–31). The appropriate threshold value was established through the construction of the ROC curve and the utilization of the Youden index. In this study, a CIN score threshold of 13,563 was identified as the most effective value for discriminating between the CIN low and CIN high groups in predicting SLNM in breast cancer. Specifically, 13 samples were categorized as belonging to the CIN low group, while the remaining 16 samples were assigned to the CIN high group. As shown in **Figure 2D**, the CIN score of the CIN high group (22,226.203 ± 6,831.045) was higher than that of the CIN low group (8,813.996 ± 2,428.643), with a statistically significant difference observed (P< 0.05).

### Associations between the general condition of patients and CIN score

**Table 2** demonstrates that the CIN score exhibited a statistically significant association with SLNM in breast cancer (*P*=0.011), while no statistically significant associations were identified with age, tumor size, histological grade, Ki67, ER, PR, HER2, molecular subtype, and chromosome arm changes. The correlation coefficient Cramer’s V between CIN score and SLNM was calculated to be 0.506 (*P*=0.011), indicating a relatively strong association (**Table 3**). We proceeded to examine the correlation between CIN score and SLNM based on these findings.

**Table 2:** Single parameter analysis of multiple clinicopathological factors between CIN High and CIN Low groups.

Variable	CIN score		χ^2^	P value
	Low (n=13)	High (n=16)		
Age			–	0.270
≤60	6 (46.2)	4 (25.0)		
>60	7 (53.8)	12 (75.0)		
Tumor size/cm			–	1.000
≤2.5	9 (69.2)	12 (75.0)		
>2.5	4 (30.8)	4 (25.0)		
Histologic grade			1.062	0.936
1	3 (23.1)	2 (12.5)		
2	5 (38.5)	8 (50.0)		
3	1 (7.7)	1 (6.3)		
Other	4 (30.8)	5 (31.3)		
ER			–	0.632
Positive	10 (76.9)	14 (87.5)		
Negative	3 (23.1)	2 (12.5)		
PR			–	0.667
Positive	9 (69.2)	13 (81.3)		
Negative	4 (30.8)	3 (18.8)		
HER2			–	0.232
Positive	0 (0.0)	3 (18.8)		
Negative	13 (100.0)	13 (81.3)		
Ki67			–	0.715
<14%	6 (46.2)	9 (56.3)		
≥14%	7 (53.8)	7 (43.8)		
Molecular type			2.916	0.417
Luminal A	4 (30.8)	8 (50.0)		
Luminal B	6 (46.2)	6 (37.5)		
Over-HER2	0 (0.0)	1 (6.3)		
TNBC	3 (23.1)	1 (6.3)		
changes of CA			–	0.061
Yes	3 (23.1)	10 (62.5)		
No	10 (76.9)	6 (37.5)		
SLNM			–	0.011
Yes	8 (61.5)	16 (100.0)		
No	5 (38.5)	0 (0.0)		

- calculated by Fisher’s exact test.

CA, Chromosome arms; TNBC, Triple Negative Breast Cancer; Over-HER2, HER2 overexpression.

**Table 3:** Correlation analysis of clinicopathological factors between CIN high and low groups.

Variable	CIN score	
	Cramer's V	P value
Age	0.221	0.270
Tumor size/cm	0.064	1.000
Histologic grade	0.155	0.936
ER	0.139	0.632
PR	0.140	0.667
HER2	0.306	0.232
Ki67	0.100	0.715
Molecular type	0.325	0.417
changes of CA	0.394	0.061
SLNM	0.506	0.011

### The predictive value of CIN score for SLNM in breast cancer before operation

It is imperative to investigate the potential correlation between the patient’s general conditions and SLNM while controlling for confounding variables to enhance the validity and precision of the research.

As shown in **Table 4**, SLNM of breast cancer was correlated with the CIN score (*P*=0.011) and chromosome arm changes (*P*=0.048) but not with age, tumor size, histological grade, ER, PR, HER2, Ki67, or molecular subtype. The correlation coefficient Cramer’s V for SLNM and CIN score was 0.506 (*P*=0.011), indicating a relatively strong association. Conversely, the Cramer’s V for SLNM and chromosome arm changes was 0.411 (*P*=0.027), suggesting a statistically noteworthy but relatively weak correlation (**Table 5**). Based on the findings from the aforementioned analysis, variables with statistical differences — CIN score and chromosome arm changes — were further analyzed using univariate binary logistic regression analysis. As shown in **Table 6**, the CIN score was an important factor affecting SLNM of breast cancer (odds ratio (OR): 4.036, 95%CI: 1.015–16.047, *P*=0.048). No statistical significance was observed between chromosome arm changes and SLNM.

**Table 4:** Single parameter analysis of multiple clinicopathological factors between metastasis and non-metastasis groups.

Variable	Sentinel lymph node metastasis		χ^2^	P value
	No (n=5)	Yes (n=24)		
Age			–	0.633
≤60	1 (20.0)	9 (37.5)		
>60	4 (80.0)	15 (62.5)		
Tumor size/cm			–	0.283
≤2.5	5 (100.0)	16 (66.7)		
>2.5	0 (0.0)	8 (33.3)		
Histologic grade			2.789	0.412
1	1 (20.0)	4 (16.7)		
2	1 (20.0)	12 (50.0)		
3	0 (0.0)	2 (8.3)		
Other	3 (60.0)	6 (25.0)		
ER			–	0.195
Positive	3 (60.0)	21 (87.5)		
Negative	2 (40.0)	3 (12.5)		
PR			–	0.075
Positive	2 (40.0)	20 (83.3)		
Negative	3 (60.0)	4 (16.7)		
HER2			–	1.000
Positive	0 (0.0)	3 (12.5)		
Negative	5 (100.0)	21 (87.5)		
Ki67			–	1.000
<14%	3 (60.0)	12 (50.0)		
≥14%	2 (40.0)	12 (50.0)		
Molecular type			3.776	0.331
Luminal A	1 (20.0)	11 (45.8)		
Luminal B	2 (40.0)	10 (41.7)		
Over-HER2	0 (0.0)	1 (4.2)		
TNBC	2 (40.0)	2 (8.3)		
changes of CA			–	0.048
Yes	0 (0.0)	13 (54.2)		
No	5 (100.0)	11 (45.8)		
CIN score			–	0.011
High	0 (0.0)	16 (66.7)		
Low	5 (100.0)	8 (33.3)		

- calculated by Fisher’s exact test.

CA, Chromosome arms; TNBC, Triple Negative Breast Cancer; Over-HER2, HER2 overexpression.

**Table 5:** Correlation analysis of clinicopathological factors between the metastatic group and the non-metastatic group.

Variable	Sentinel lymph node metastasis	
	Cramer's V	P value
Age	0.139	0.633
Tumor size/cm	0.282	0.283
Histologic grade	0.317	0.482
ER	0.275	0.195
PR	0.383	0.075
HER2	0.155	0.622
Ki67	0.076	1.000
Molecular type	0.366	0.431
changes of CA	0.411	0.027
CIN score	0.506	0.011

**Table 6:** Univariate binary logistic regression analysis of influencing SLNM in breast cancer.

Variable	OR(95%CI)	95%CI	P value
changes of CA	1.3618E-9	–	0.999
CIN score^a^	4.036	1.015, 16.047	0.048

a CIN score: the data were log10 transformed and standardized to improve the prediction ability of the model and enhance the stability of the algorithm.

**Figure 3:**
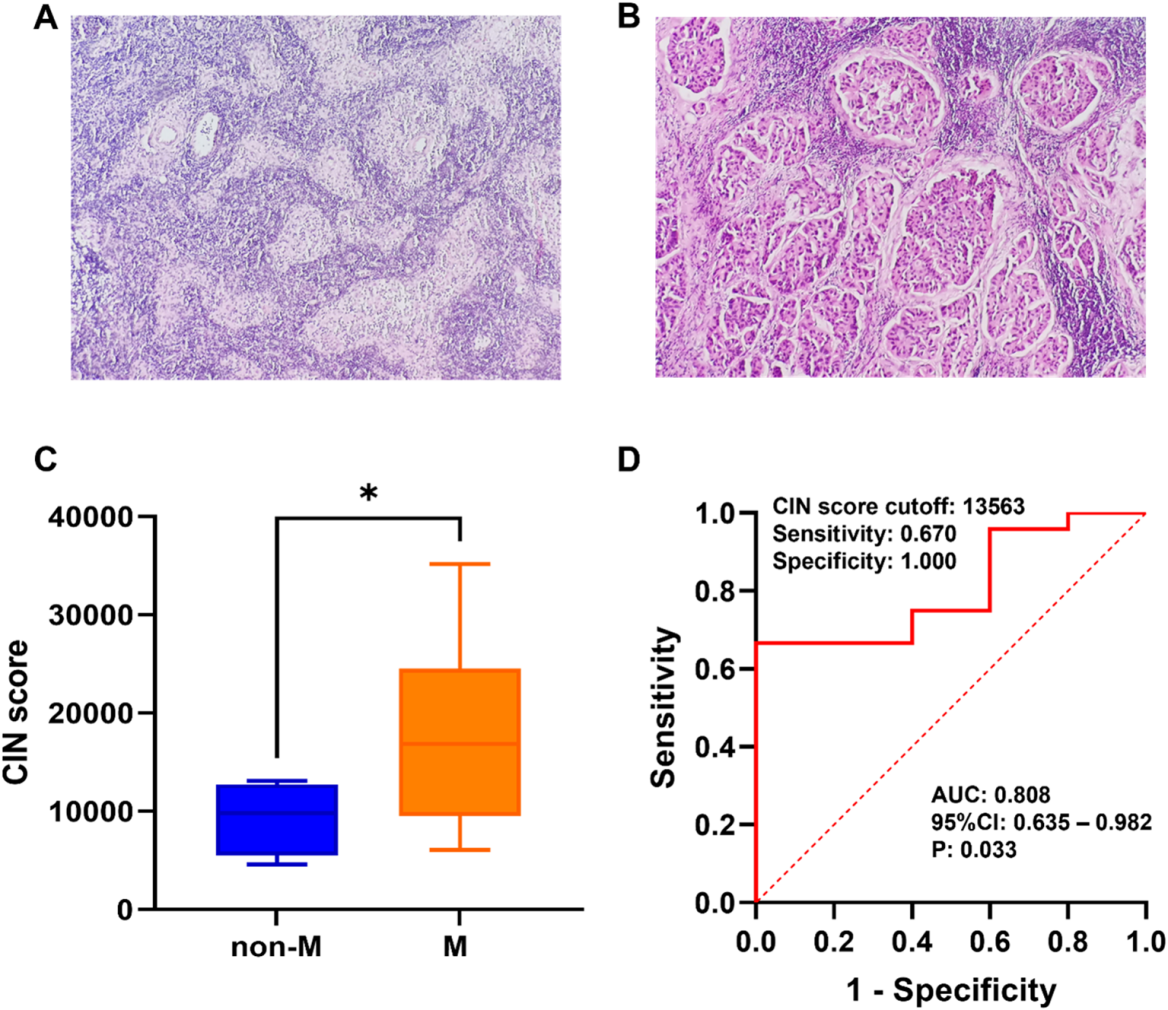
**(A)** Microscopic schematic of SLN in the non-metastatic group. **(B)** Microscopic schematic of SLN in the metastatic group. **(C)** Comparison of CIN scores between the SLN metastasis group (M) and non-metastasis group (non-M). **(D)** Performance of CIN score in preoperative prediction of SLNM in breast cancer. *P < 0.05.

Based on the findings of SLNB, patients were grouped according to the existence or lack of metastasis. Microscopic images depicting SLN metastasis and non-metastasis are presented in **Figures 3A, B**. As shown in **Figure 3C**, the CIN score of the metastasis group (17,665.055 ± 8,630.691) was notably greater compared with that of the non-metastasis group (9,247.973 ± 3,692.873), demonstrating a significant difference (*P*=0.044). The ROC curve was utilized to assess the efficacy of CIN in predicting SLNM in breast cancer. At a CIN score cut-off of 13,563, the AUC was determined to be 0.808 (95%CI: 0.635–0.982, *P*=0.033), with a sensitivity of 0.670 and a specificity of 1.000 (**Figure 3D**). The results indicate that the CIN score demonstrates favorable diagnostic performance in predicting SLNM.

## Discussion

Prior research has indicated that 89% of patients with invasive breast cancer exhibit CIN, implying its potential utility in diagnosis and treatment (7). Furthermore, LPWGS offers advantages, such as comprehensive genome coverage and sensitivity for detecting significant DNA alterations (11), and it has been utilized to evaluate the connection between overall genome status and the diagnosis, recurrence, and prognosis of tumors (11, 27, 32, 33). Our study identified CIN in patients with breast cancer using LPWGS and assessed the diagnostic utility of CIN for the preoperative prediction of SLNM. Our analysis revealed a significant correlation between the CIN score and SLNM (P=0.011), indicating that the CIN score is a crucial determinant of SLNM in patients with breast cancer. The OR for this association was 4.036, with a 95% CI ranging from 1.015 to 16.047, and a P-value of 0.048. The CIN score of metastasis group (17 665.055 ± 8 630.691) was significantly higher than that of non-metastasis group (9 247.973 ± 3 692.873) (P=0.044). At a CIN score cut-off of 13,563, the AUC for CIN in predicting SLNM was 0.808, indicating a relatively high level of diagnostic accuracy. Specifically, the diagnostic sensitivity of CIN reached 67.0%, with a specificity of 100%, demonstrating significant diagnostic value. Furthermore, a comparative analysis with the non-metastatic group revealed that the copy number coverage chart exhibited significant variations in chromosome copy numbers within the metastatic group. Notably, chromosomal aberrations were frequently observed on chromosomes 1, 7, 8, 12, 13, 16, 17, 18, and 20. In clinical practice, CIN status can be identified by LPWGS after precise biopsy sampling. Research suggests that CIN could be widely utilized in the preoperative assessment of axillary lymph node status.

The degree of CIN in metastatic breast cancer is greater than that in primary breast cancer (9). Our research demonstrated that the CIN score significantly influenced the likelihood of SLNM in patients with breast cancer (OR: 4.036, 95%CI: 1.015-16.047, P =0.048) (**Table 6**). Individuals in the CIN high group exhibited a greater risk of SLNM than those in the CIN low group. Moreover, the AUC for CIN in predicting SLNM was 0.808, indicating a relatively high level of diagnostic accuracy (**Figure 3D**). These findings indicate that assessing CIN through LPWGS could serve as a valuable method to predict SLNM in patients with breast cancer. Bakhoum et al. (23) discovered that CIN facilitates metastasis by enabling an independent reaction of tumor cells to cytoplasmic DNA. Mistakes in the segregation of chromosomes lead to an accumulation of micronuclei, which release genomic DNA into the cytoplasm. This process triggers the stimulation of the cGAS-STING (cGMP-AMP synthetase stimulator of IFN genes) cytoplasm DNA-sensing pathway and subsequent noncanonical NF-κB signaling (23). This signaling results in upregulating inflammatory and EMT genes essential to metastasis. The authors’ demonstration of the significant delay in metastasis through the inhibition of CIN, as well as the promotion of cell invasion and metastasis through persistent segregation errors, establishes a causal relationship between CIN and metastasis. This suggests that CIN acts as the primary driver of metastasis (23). Our study extends prior research findings and is dedicated to the translation of pre-clinical research into practical applications. Utilizing this evaluative methodology, we aim to enhance the accuracy of preoperative diagnosis of SLNM in patients with breast cancer, thereby contributing to the advancement of more personalized and efficacious treatment strategies.

In the present study, despite the inclusion of only two cases of triple-positive breast cancer (TPBC)—characterized by the concurrent positivity of ER, PR, and HER2, and classified as the luminal B molecular subtype—these patients demonstrated exceptionally high CIN scores and exhibited significant chromosomal abnormalities (**Figure 2C**). Luminal B type tumors constitute approximately 15-20% of breast cancer cases and, in comparison to luminal A type, generally display a more aggressive phenotype, elevated histological grade, and less favorable prognosis (34, 35). Camargo et al. (36) identified that patients with luminal B type breast cancer frequently exhibit moderate levels of CIN and stable aneuploidy, both of which are correlated with lymphovascular invasion. Notably, within their cohort, the sole patient characterized as TNBC demonstrated a high degree of CIN (36). Regarding chromosomal alterations, existing studies have demonstrated that in ER+ and HER2+ breast cancers, recurrent arm-level events observed in metastatic tumors, as compared to primary tumors, are predominantly clonal (21). Furthermore, Tousled-like kinase 2 (TLK2), a cell cycle-regulating kinase, exhibits a higher frequency of amplification in luminal B breast cancer (37). The overexpression of TLK2 has been shown to augment the invasiveness of breast cancer cells (37). However, the limited sample size in this study precluded a more detailed examination of the specific associations underlying these observations. Future research should aim to increase the sample size to facilitate a more comprehensive investigation of the relationship between CIN and TPBC, as well as to elucidate the potential molecular mechanisms involved.

Prior research has demonstrated that breast cancer genomes typically display tetraploidy or near-triploidy (38), along with quantitative and intricate structural chromosomal abnormalities and alterations in fragment copy numbers (39). Research has identified chromosomal deletions in breast cancer, notably in regions, such as 1p, 3p, 8p, 11q, 13q, 16q, 17p, and 17q, while amplification is prevalent in chromosome 1q, 8q, 11q, and 17q regions (13). For example, Goh et al. (40) demonstrated that chromosomal 1q21.3 amplification is prevalent in breast cancer, occurring in 10–30% of initial lesions and over 70% of recurrent and metastatic lesions. Their analysis revealed a strong association between this chromosomal amplification and aberrant gene expression, suggesting a potential involvement in the metastatic progression of breast cancer (40). Similarly, our study indicated a higher prevalence of gains in chromosome arms 1q and 8q, as well as losses in chromosome arms 12q, 16q, 17p, and 17q within the metastatic group (**Figures 2A–C**). Furthermore, alterations in specific genes may initiate or enhance CIN, thereby influencing the metastatic progression of cancer (7). For instance, mutations in the TP53 gene on 17p and the C-myc gene on 8q have the potential to induce CIN (41–43). Huo et al. (44) demonstrated that SIRT7 on 17q facilitates breast cancer metastasis through the SIRT7/LAP2α signaling pathway. Similarly, the upregulation of MASTL on 10p in breast cancer is intricately linked to the progression of the disease (45, 46). Research conducted by Rogers et al. provides additional evidence that the upregulation of MASTL may be crucial in breast cancer progression. This upregulation results in disruptions in chromosome segregation, and an increase in micronucleus formation by disrupting the timing of mitotic exit, ultimately exacerbating CIN and facilitating the invasion and metastasis of tumors (47). Due to constraints imposed by the sample size, further investigation is required to elucidate the precise relationship between alterations in these specific chromosome arms and SLNM in breast cancer.

Our study primarily examined the correlation between SLNM and CIN and assessed the preoperative predictive efficacy of CIN for SLNM in breast cancer. However, the study has some limitations. First, the study’s focus was primarily on clinicopathological features and genomic testing for breast cancer. However, it is important to note that imaging techniques can also offer crucial information on the biological characteristics of breast cancer and the metastatic process. Furthermore, this study’s retrospective nature, limited sample size, and challenges in obtaining comprehensive clinical data from patients may have impacted the outcomes of the study.

In summary, the current study utilized the LPWGS to quantify CIN and identified it as a significant factor influencing SLNM in breast cancer. CIN demonstrates promising diagnostic utility in predicting SLNM and may serve as a helpful indicator for the preoperative assessment of SLNM in breast cancer. This discovery offers a novel approach for clinicians to assess axillary lymph node status preoperatively, enhancing diagnostic accuracy and advancing breast cancer diagnosis and treatment. Future research should consider enlarging sample sizes and conducting prospective multicenter studies to further assess the diagnostic efficacy of CIN in predicting SLNM in breast cancer prior to surgery.

## Data Availability

The data presented in the study are deposited in the Genome Sequence Archive repository, https://ngdc.cncb.ac.cn/gsa-human/browse/HRA008309.
